# Combining prostate health index and multiparametric magnetic resonance imaging in the diagnosis of clinically significant prostate cancer in an Asian population

**DOI:** 10.1007/s00345-019-02889-2

**Published:** 2019-08-22

**Authors:** Po-Fan Hsieh, Wei-Juan Li, Wei-Ching Lin, Han Chang, Chao-Hsiang Chang, Chi-Ping Huang, Chi-Rei Yang, Wen-Chi Chen, Yi-Huei Chang, Hsi-Chin Wu

**Affiliations:** 1 Department of Urology, China Medical University Hospital, No. 2, Yu-Der Rd, Taichung, Taiwan; 2 School of Medicine, China Medical University, Taichung, Taiwan; 3 Graduate Institute of Biomedical Sciences, School of Medicine, China Medical University, Taichung, Taiwan; 4 Department of Radiology, China Medical University Hospital, Taichung, Taiwan; 5 Department of Pathology, School of Medicine, China Medical University, Taichung, Taiwan; 6 Department of Urology, China Medical University Beigang Hospital, Beigang, Yunlin Taiwan

**Keywords:** Prostate cancer detection, Prostate health index, mpMRI, PI-RADS

## Abstract

**Objective:**

To evaluate the practicability of combining prostate health index (PHI) and multiparametric magnetic resonance imaging (mpMRI) for the detection of clinically significant prostate cancer (csPC) in an Asian population.

**Patients and methods:**

We prospectively enrolled patients who underwent prostate biopsy due to elevated serum prostate-specific antigen (PSA > 4 ng/mL) and/or abnormal digital rectal examination in a tertiary referral center. Before prostate biopsy, the serum samples were tested for PSA, free PSA, and p2PSA to calculate PHI. Besides, mpMRI was performed using a 3-T scanner and reported in the Prostate Imaging Reporting and Data System version 2 (PI-RADS v2). The diagnostic performance of PHI, mpMRI, and combination of both was assessed.

**Result:**

Among 102 subjects, 39 (38.2%) were diagnosed with PC, including 24 (23.5%) with csPC (Gleason ≥ 7). By the threshold of PI-RADS ≥ 3, the sensitivity, specificity, positive predictive value (PPV), and negative predictive value (NPV) to predict csPC were 100%, 44.9%, 35.8%, and 100%, respectively. By the threshold of PHI ≥ 30, the sensitivity, specificity, PPV, and NPV to predict csPC were 91.7%, 43.6%, 33.3%, and 94.4%, respectively. The area under the receiver operator characteristic curve of combining PHI and mpMRI was greater than that of PHI alone (0.873 vs. 0.735, *p* = 0.002) and mpMRI alone (0.873 vs. 0.830, *p* = 0.035). If biopsy was restricted to patients with PI-RADS 5 as well as PI-RADS 3 or 4 and PHI ≥ 30, 50% of biopsy could be avoided with one csPC patient being missed.

**Conclusion:**

The combination of PHI and mpMRI had higher accuracy for detection of csPC compared with PHI or mpMRI alone in an Asian population.

## Introduction

With the aging society and serum prostate-specific antigen (PSA) screening, the incidence of prostate cancer (PC) increased in recent decades. Worldwide PC is the most prevalent noncutaneous malignancy as well as the third leading cause of cancer death in males [1]. Although the incidence of PC is much lower in Asia than in Western countries, it has been growing especially in Eastern Asian countries [2]. The possible causes included the Westernized diet and development of cancer registration systems.

Traditionally, PSA has been used a biomarker of PC. However, the accuracy of PSA to detect clinically significant prostate cancer (csPC) was not desirable. In a large prospective study, the positive predictive value (PPV) of PC detection was only 32% for PSA [3]. The low specificity of PSA might contribute to unnecessary prostate biopsy and the exposure of complications including bleeding, pain, or sepsis. Over the past decade, prostate health index (PHI), measured by [− 2]proprostate-specific antigen (p2PSA), free PSA, and total PSA, was developed and showed promising outcomes as a predictive biomarker of positive prostate biopsy [4]. PHI outperformed total or free PSA for the detection of csPC both in the initial and repeat biopsy setting, and it could also decrease unnecessary prostate biopsies [5, 6].

On the other hand, the role of imaging assessment prior to biopsy cannot be overemphasized [7]. Multiparametric magnetic Resonance Imaging (mpMRI) emerged and was considered as a useful tool to identify the suspicious lesion and to guide prostate biopsy [8, 9]. Currently, the Prostate Imaging Reporting and Data System version 2 (PI-RADS v2) released by an international collaboration of the American College of Radiology (ACR) and European Society of Uroradiology (ESUR) in 2015 is commonly used as a standardized reporting system of mpMRI [10]. The excellent diagnostic performance of mpMRI reporting in PI-RADS v2 for csPC has been reported consistently. In a meta-analysis, the pooled sensitivity was 0.89 (95% CI 0.86–0.92) and specificity was 0.73 (95% CI 0.6–0.83) [11]. In that study, the updated PI-RADS v2 also showed significant improvement in cancer detection rate compared with the original PI-RADS v1. Another prospective multicenter study reported that using mpMRI allowed 27% of patients to avoid an unnecessary prostate biopsy [12].

Despite the favorable diagnostic accuracy, PHI is not yet widely available. Pre-biopsy mpMRI might not be covered by health insurance in many countries, either. To the best of our knowledge, few articles evaluate the predictive ability of the combination of PHI and mpMRI in patients with suspicion of PC [13–15]. Therefore, we conducted this study to evaluate the feasibility of integrating PHI and mpMRI for the detection of csPC in an Asian population.

## Materials and methods

### Study population

From August 2016 to November 2018, after obtaining informed consent, we enrolled patients who were more than 40 years and underwent prostate biopsy for suspicious PC due to elevated serum PSA level (PSA > 4 ng/mL) and/or abnormal findings on DRE in a tertiary referral center. All patients had received PHI test as well as prostate mpMRI before biopsy. The results of PHI or mpMRI were not disclosed to the patients; they did not interfere with the decision of biopsy, either. The exclusion criteria were patients who had histories of PC, bacterial prostatitis in 3 months before biopsy, use of 5-alpha reductase inhibitors, or inability/unwillingness to sign informed consent. After institutional review board approval, the patients’ clinical characteristics and biopsy results were prospectively recorded and analyzed. We followed the Standards of Reporting for MRI-Targeted Biopsy Studies (START) guidelines to report mpMRI and the biopsy results [16].

### Laboratory methods

We tested PSA parameters including total PSA, free PSA, and p2PSA from serum samples collected before prostate biopsy. After withdrawal of blood, it was centrifuged within 3 h, and frozen at − 20 to − 80 ℃ until analysis [17]. We used a Beckman Coulter DxI 800 Immunoassay System to determine PHI using the formula PHI = (p2PSA/free PSA) × √PSA [18].

### Magnetic resonance imaging protocol

Multiparametric MRI was performed using a 3-T scanner (Signa HDxt, GE Healthcare, Milwaukee, WI) with a four-channel high definition (HD) cardiac array coil. The scanning protocol of mpMRI included T2-weighted imaging (T2WI), diffusion-weighted imaging (DWI), and dynamic contrast enhanced (DCE). DWI was acquired with *b* values of 0 and 1000 s/mm^2^, and an apparent diffusion coefficient (ADC) map was generated.

All mpMRI was interpreted by a single uroradiologist (W. C. L.) who had 10 years of prostate MRI experience. Each suspicious cancerous lesion was scored according to PI-RADS v2 and marked on a picture archiving and communication system workstation (Infinitt Healthcare, Phillipsburg, NJ) [10].

### Biopsy protocol

Prostate biopsy was done, while the patients were under intravenous general anesthesia. One urologist (P. F. H. or Y. H.C.) who is blinded to the PHI results reviewed the mpMRI and identified the most suspicious lesions from those with PI-RADS ≥ 3 as the target lesions (maximum three target lesions per patient). Then, cognitive registration targeted biopsy (TB) was performed, followed by systematic biopsy (SB), using a biplane TRUS probe (BK Medical, Transducer 8818) and an 18-G needle. For each target lesion, at least 2 cores were sampled, and at least 12 cores were sampled systematically from bilateral peripheral zones of prostate.

### Histopathological analysis

The biopsy specimens were interpreted by an experienced uropathologist (H. C.). PC was graded in accordance with 2014 International Society of Urological Pathology Consensus Conference guidelines [19]. In detail, grade group (GG) 1 equals Gleason Score (GS) ≤ 3 + 3; GG 2 equals GS 3 + 4; GG 3 equals GS 4 + 3; GG 4 equals GS 8; GG 5 equals GS 9–10. We defined csPC as PC with GG ≥ 2 [20].

### Statistical analysis

The continuous variables were reported as median (IQR), and the categorical variables were reported as proportions. Between csPC and non-clinically significant cancer (non-csPC; including GG 1 cancer or no cancer) groups, these variables were compared using student *t* test or Chi-square test, as appropriate. Univariate logistic regression analysis was performed to determine the association between each covariate and csPC. According to a meta-analysis of PHI in Asian population, we chose PHI ≥ 30 as a diagnostic threshold [21]. The sensitivity, specificity, PPV, and negative predictive value (NPV) of PHI and mpMRI were calculated for the detection of csPC. The association between PHI and biopsy tumor burden was also evaluated. Finally, the diagnostic performance of PHI, mpMRI, and combination of both were assessed using receiver operating characteristic (ROC) curve analysis. All statistical analyses were carried out using SPSS version 22 (IBM Corp, Armonk, NY, USA), assuming a two-sided test with a 5% level of significance. The areas under curve (AUC) were compared using DeLong’s method [22].

## Results

There were 109 patients undergoing prostate biopsy. Four patients were excluded because of lack of DCE or DWI in the mpMRI protocol or poor quality of mpMRI. Three patients were excluded because of insufficient cores of SB or lack of TB for the suspicious lesions on mpMRI. Finally, 102 patients were enrolled in the study cohort. Table 1 shows the characteristics of the study population. Among the 102 patients, 24 (23.5%) were diagnosed with csPC and 15 (14.7%) were diagnosed with GG 1 PC. Patients with csPC were less likely to have the previous negative biopsy than those with non-csPC (20.8% vs. 37.2%, *p* < 0.001). The median PSA and PHI were significantly higher in patients with csPC (9.05 vs. 7.55, *p* = 0.02 and 46.79 vs. 32.99, *p* < 0.001, respectively). All patients with csPC had PI-RADS ≥ 3 lesions on mpMRI, while only 55.1% of patients with non-csPC had PI-RADS ≥ 3 lesions on mpMRI (*p* < 0.001).

**Table 1:** Patient characteristics

	Total (*N *= 102)	CsPC^b^ (*N *= 24)	Non-csPC (*N *= 78)		*p* value^c^
			GG1 ca (*N *= 15)	No ca (*N *= 63)	
Age, years	65.5 (60–70)	67 (60.75–73)	64.5 (60–70)		0.950
			70 (65.5–76.5)	63 (60–68)	
Abnormal DRE, *N* (%)	38 (37.3)	13 (54.2)	25 (32.1)		0.083
			9 (60.0)	16 (25.4)	
Previous negative biopsy, *N* (%)	34 (33.3)	5 (20.8)	29 (37.2)		< 0.001
			5 (33.3)	24 (38.1)	
PSA, ng/mL	7.78 (6.12–11.8)	9.05 (6.58–12.31)	7.55 (6.01–11.33)		0.02
			7.33 (5.95–10.26)	7.64 (6.05–12.05)	
% free PSA	17 (13.0–22.6)	13 (10.0–17.4)	19.1 (14.4–25.4)		0.136
			19.2 (15.4–31.3)	19.1 (14.1–24.1)	
PHI	39.67 (27.63–49.08)	46.79 (35.12–82.76)	32.99 (25.54–44.95)		< 0.001
			43.17 (27.0–55.81)	31.28 (25.52–44.57)	
Positive mpMRI^a^, *N* (%)	67 (65.7)	24 (100)	43 (55.1)		< 0.001
			14 (93.3)	29 (46.0)	

All values given as number (percentage, %) or median (IQR)

*N* number, *csPC* clinically significant prostate cancer, *non-csPC* non-clinically significant prostate, *GG* grade group, *ca* cancer, *DRE* digital rectal examination, *PSA* prostate-specific antigen, *PHI* prostate health index, *mpMRI* multiparametric magnetic resonance imaging

^a^Positive mpMRI is defined as PI-RADS ≥ 3

^b^csPC is defined as GG ≥ 2 prostate cancer

^c^*p* value shows the significance between csPC and non-csPC

Table 2 shows the univariate logistic regression analysis for the prediction of PC. For total PC, age, DRE, PHI, and mpMRI were significant predictors (*p* = 0.008, 0.002, 0.001, and < 0.001, respectively). For csPC, % free PSA, PHI, and mpMRI were significant predictors (*p* = 0.003, 0.001, and < 0.001, respectively). By the threshold of PI-RADS ≥ 3, the sensitivity, specificity, PPV, and NPV of mpMRI to predict csPC were 100%, 44.9%, 35.8%, and 100%, respectively. By the threshold of PHI ≥ 30, the sensitivity, specificity, PPV, and NPV of PHI to predict csPC were 91.7%, 43.6%, 33.3%, and 94.4%, respectively. In addition, PC patient with PHI ≥ 30 seemed to have more positive biopsy cores (4 vs. 2), maximum percentage of cancer in positive cores (27.5% vs. 16.5%), and maximum cancer core length (4.5 mm vs. 2.48 mm) than those with PHI < 30, but none of them reached clinically significant difference (Table 3).

**Table 2:** Univariate logistic regression analysis for the prediction of total and clinically significant prostate cancer (csPC)

	Total PC		csPC	
	OR (95% CI)	*p* value	OR (95 CI)	*p* value
Age	1.077 (1.020–1.138)	0.008	1.033 (0.977–1.092)	0.251
Abnormal DRE	3.801 (1.625–8.894)	0.002	2.505 (0.985–6.370)	0.054
PNB	0.560 (0.232–1.352)	0.197	0.445 (0.150–1.318)	0.144
PSA	1.032 (0.986–1.079)	0.179	1.033 (0.991–1.078)	0.129
% free PSA	0.292 (0.002–6.530)	0.292	< 0.001 (< 0.001–0.017)	0.003
PHI	1.038 (1.015–1.0561)	0.001	1.041 (1.017–1.065)	0.001
mpMRI	5.303 (2.703–10.401)	< 0.001	5.150 (2.333–11.370)	< 0.001

*PC* prostate cancer, *csPC* clinically significant prostate cancer, *DRE* digital rectal examination, *PNB* previous negative biopsy, *PSA* prostate-specific antigen, *PHI* prostate health index, *mpMRI* multiparametric magnetic resonance imaging

**Table 3:** Association between PHI and tumor burden

	PHI < 30	PHI ≥ 30	*p* value
Number of positive cores, *N*	2	4	0.062
Maximum percentage of cancer in positive cores, %	16.5	27.5	0.239
Maximum cancer core length, mm	2.48	4.5	0.434

All values given as median

*N* number, *PHI* prostate health index

**Fig. 1:**
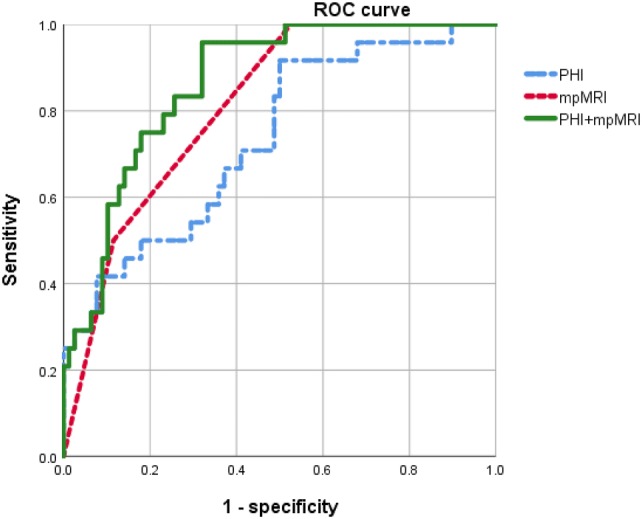
Receiver-operating characteristic (ROC) curve analysis for PHI, mpMRI, and combination of both to predict clinically significant prostate cancer. The area under curve (AUC) of PHI, mpMRI, and combination of PHI and mpMRI were 0.735 (95% CI 0.6194–0.8497), 0.830 (95% CI 0.7598–0.9004), and 0.873 (95% CI 0.8050–0.9407). PHI, prostate health index; mpMRI, multiparametric magnetic resonance imaging

On ROC analysis, the AUC of PHI, mpMRI, and combination of PHI and mpMRI was 0.735 (95% CI 0.6194–0.8497), 0.830 (95% CI 0.7598–0.9004), and 0.873 (95% CI 0.8050–0.9407), respectively (Fig. 1). The AUC of combination of PHI and mpMRI was significantly higher than that of PHI alone (*p* = 0.002) and mpMRI alone (*p* = 0.035).

**Fig. 2:**
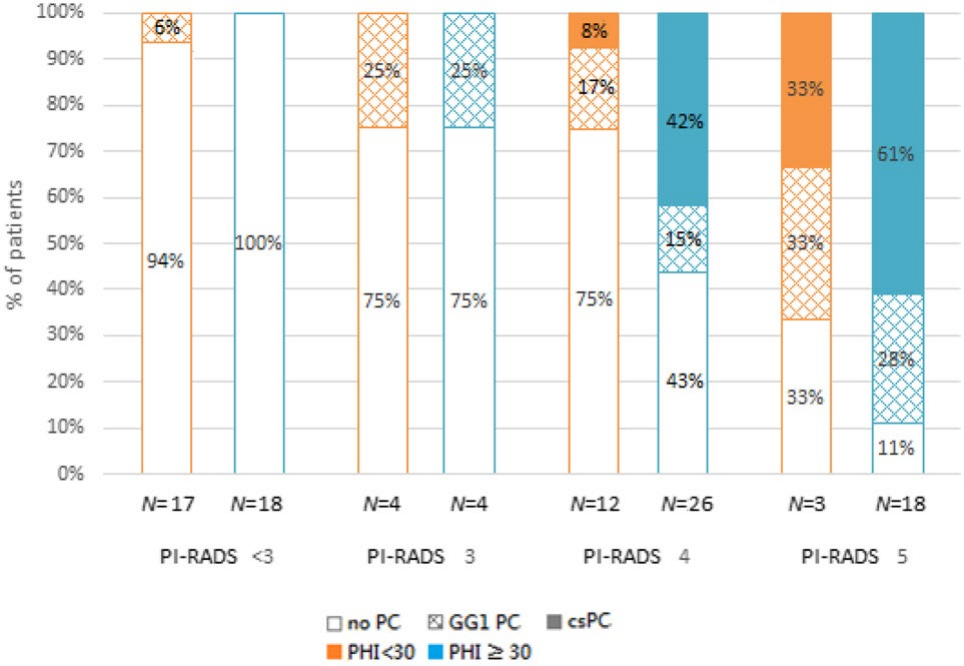
Pathological outcomes subcategorized by PHI and PI-RADS score. *N* number, *PC* prostate cancer, *csPC* clinically significant prostate cancer, *GG* grade group, *ca* cancer, *PI-RADS* Prostate Imaging Reporting and Data System, *PHI* prostate health index

Figure 2 shows the distribution of biopsy outcomes subcategorized PHI and PI-RADS score. If biopsy was restricted to patients with PI-RADS ≥ 3, 34.3% of biopsy could be avoided. If biopsy was restricted to patients with PHI ≥ 30, 35.3% of biopsy could be avoided, but two csPC patients were missed. Furthermore, if biopsy was restricted to patients with PI-RADS 5 as well as PI-RADS 3 or 4 and PHI ≥ 30, up to 50% of biopsy could be avoided with only one csPC patient being missed.

## Discussion

This is the first prospective study to evaluate PHI, mpMRI, and combination of both to predict csPC before prostate biopsy in an Asian population. We found that both PHI and mpMRI had a high NPV and could independently predict biopsy outcome. If we combined PHI and mpMRI, the area under the ROC curve is even higher than that of PHI or mpMRI alone and more unnecessary biopsy could be avoided.

PHI and mpMRI have been suggested as biomarkers before biopsy to identify csPC and reduce unnecessary biopsy [12, 23, 24]. In recent years, some studies were conducted to integrate these serum and imaging biomarkers. Gnanapragasam et al. proposed the first evidence of the complementary role for PHI and mpMRI [13]. In the repeat biopsy setting with a transperineal approach, they found that the combination of PHI and mpMRI improved predictive performance for overall and clinically significant (GS ≥ 7) cancer detection compared with mpMRI alone (AUC 0.75 vs. 0.69). Furthermore, applying a PHI threshold of ≥ 35 among men with negative mpMRI could spare 42% of biopsies, while only missing a single low-volume csPC. However, in their series, DCE was not adopted in the imaging protocol, and the interpretation of mpMRI was not based on PI-RADS v2 either. Therefore, it remained inconclusive to apply their results in the initial biopsy setting, especially in the era of updated PI-RADS v2.

In a real-world practice, Tosoian et al. also provided the complementary information of PHI to mpMRI [14]. They observed that no GG ≥ 2 PC was diagnosed in 15 men with PHI < 27 and PI-RADS ≤ 3. However, in their study, PHI and mpMRI were arranged based on physicians’ clinical judgment rather than being obtained in all patients. On the contrary, in our series, PHI and mpMRI were ordered for every patient, and mpMRI were interpreted using updated PI-RADS v2. More importantly, prostate biopsy was done for all patients, regardless of mpMRI findings or PHI level. Therefore, we can see more clearly the impact of PHI and mpMRI on cancer detection rate. Besides, we took racial difference of PHI in consideration. In NCCN guideline of PC early detection, PHI > 35 was suggested to estimate high-grade cancer [25]. However, in some Eastern Asian studies, at sensitivity of 90%, the cutoff of PHI was set ranging from 24.9 to 32 [26–28]. A recent multicenter study recommended PHI > 30 to predict high-grade (GS ≥ 7) cancer in Asian men, whereas the threshold should be > 40 for European men [21]. In Table 4, we compared the diagnostic performance of various PHI cutoffs. Although up to 44.1% of biopsy could be avoided by the criteria of PHI ≥ 35, 20.8% of csPC were missed. On the other hand, PHI ≥ 30 served as a better cutoff balancing the percentage of biopsy avoided and percentage of csPC missed. Furthermore, the combination of PHI and mpMRI could spare more biopsy than criteria using PHI or mpMRI alone (50% vs. 35.3% and 50% vs. 34.3%, both *p* < 0.001).

**Table 4:** Diagnostic performance for clinically significant prostate cancer by different PHI cutoffs

	Sensitivity	Specificity	PPV	NPV	Biopsy criteria by PHI alone		Biopsy criteria by PHI and mpMRI^a^	
					% biopsy avoided	% csPC missed	% biopsy avoided	% csPC missed
PHI ≥ 25	95.8%	23.1%	27.7%	94.7%	18.6%	4.2%	18.6%	4.2%
PHI ≥ 30	91.7%	43.6%	33.3%	94.4%	35.3%	8.3%	50%	4.2%
PHI ≥ 35	79.2%	51.3%	33.3%	88.9%	44.1%	20.8%	53%	8.3%
PHI ≥ 40	70.8%	56.4%	33.3%	86.2%	50%	29.2%	57.8%	16.7%

*PHI* prostate health index, *PPV* positive predictive value, *NPV* negative predictive value, *mpMRI* multiparametric magnetic resonance imaging, *csPC* clinically significant prostate cancer

^a^Biopsy was restricted to patients with PI-RADS 5 as well as PI-RADS 3 or 4 and PHI ≥ cutoff

Based on the high diagnostic accuracy of mpMRI, a consensus by American Urological Association (AUA) and Society of Abdominal Radiology (SAR) suggested immediate repeat biopsy for a PI-RADS 4 or 5 lesion detected on mpMRI, and biopsy for a PI-RADS 3 lesion should not be routinely deferred [29]. However, the PPV of mpMRI is suboptimal. In a large MRI in-bore targeted biopsy study, Venderink et al. demonstrated that csPC was diagnosed in 17%, 34% and 67% of patients with PI-RADS 3, 4, and 5 lesions, respectively [30]. If they applied PSA density (PSAD) ≥ 0.15 as a cutoff, 42%, 38%, and 23% of PI-RADS 3, 4, and 5 lesions could avoid biopsy, but 6%, 23%, and 52% of csPC would be missed, respectively. In our series, the PPV of a PI-RADS ≥ 3 lesion for csPC was 35.8%. Nevertheless, if we performed biopsy in all patients with PI-RADS 5 and added PHI ≥ 30 as selection criteria in patients with PI-RADS 3 or 4, up to 50% of biopsy could be avoided with only one patient of csPC being missed. This patient underwent radical prostatectomy, and his pathological stage was T2 with GG 3. Therefore, the combination of PHI and mpMRI may be promising for pre-biopsy assessment to detect csPC and avoid unnecessary biopsy as much as possible. Finally, we propose the algorithm that mpMRI should be used a triage test for patients with clinical suspicion of PC. For PI-RADS 5 lesions, patients should proceed to prostate biopsy. For PI-RADS 4 or less lesions, PHI should be tested to optimize the decision making of prostate biopsy.

Another strength of this study is that we assessed the association between PHI and biopsy tumor burden. Numerous studies have reported the association between PHI and csPC focusing on Gleason grade [4–6]. Among patients with low-risk PC, PHI was also found to predict disease reclassification [31]. In our study, PHI in GG ≥ 2 PC was higher than that in GG 1 PC (46.79 vs. 43.17, *p* = 0.047). The biopsy tumor burden, including number of positive cores, maximum percentage of cancer in positive cores, and maximum cancer core length, was slightly higher in patients with PHI ≥ 30 compared to those with PHI < 30. Possibly, owing to limited case numbers, we failed to demonstrate a significant difference of biopsy tumor burden between these two groups. Further studies are needed to evaluate the influence of PHI on tumor burden.

Recently Druskin et al. reported the combination of mpMRI and PHI density (PHID), as retrospectively determined by the ratio of PHI and prostate volume measured on transrectal ultrasonography at biopsy, to predict biopsy outcome [32]. In a series of 104 men, PI-RADS ≥ 3 or PHI density (PHID) ≥ 0.44 could detect 100% of csPC. Nevertheless, the concept of PHID is not yet validated well nor a consensus threshold is reached [14, 26]. In addition, PHID cannot help decision making of prostate biopsy if it is calculated at the time of transrectal ultrasound-guided biopsy.

There are several limitations to this study. First, the study cohort does not represent a screening population. Patients were enrolled due to the increased likelihood of csPC based on demographic or laboratory findings in a tertiary referral center. Further studies are needed to validate the results to patients in other health-care settings and other ethnical groups. Second, the number of this cohort is limited, and it is even underpowered to separate patients into initial and repeat biopsy groups. Thus, this should be regarded as a pilot study of combining PHI and mpMRI to detect csPC. Further validation studies in biopsy naive and previous negative biopsy populations, respectively, are warranted. Third, the NPV of mpMRI in our cohort was 100%, which may also be attributed to limited case numbers. Besides, we used the combination of transrectal TB and SB as the pathological reference standard. The diagnostic accuracy analysis should be better using the transperineal template-guided mapping biopsy. Although it is debatable whether patients with negative mpMRI should proceed to SB, for young males with PSA or PHI elevation, SB may still be needed. Moreover, there was lack of correlation with radical prostatectomy or follow-up for patients with non-csPC. As a result, we could not demonstrate the proportion of pathological upgrading. Finally, in our study, TB was done with cognitive registration, of which the accuracy might be inferior to MR/US fusion platforms.

## Conclusion

The combination of PHI and mpMRI had higher accuracy for detection of csPC compared with PHI or mpMRI alone in an Asian population. Up to 50% of prostate biopsy could be avoided when biopsy was restricted to patients with PI-RADS 5 as well as PI-RADS 3 or 4 and PHI ≥ 30. External validation studies are needed to confirm the integration of PHI and mpMRI in the detection of csPC.
